# Letter to editor about prediction of sepsis among patients with major trauma using artificial intelligence: a multicenter validated cohort study

**DOI:** 10.1097/JS9.0000000000003353

**Published:** 2025-09-09

**Authors:** Yiran Yuan, Xuewen Jia, Bo Qi

**Affiliations:** Department of Intensive Care Unit, Jiangyou 903 Hospital, Jiangyou City, Mianyang City, Sichuan Province, China

*Dear Editor,*

We read with great interest the recent article titled “Prediction of sepsis among patients with major trauma using artificial intelligence: a multicenter validated cohort study”^[1]^. While the authors have addressed a timely and clinically relevant topic, we believe there are several important issues regarding the study’s rationale and methodology that warrant further discussion. The letter to the editor adheres to the 2025 TITAN Guidelines on the declaration and responsible use of AI^[2]^.

First, the authors state that current clinical tools for early sepsis prediction in ICU trauma patients are limited. However, this claim is not supported by a systematic review of prior work in this area. In recent years, multiple machine learning models have been proposed for early detection of sepsis, including those specifically developed for trauma populations or ICU settings^[3,4]^. Clarifying how the present model offers tangible improvements in interpretability, data input requirements, or prospective validation over existing tools would enhance the study’s originality.

Second, the derivation cohort was drawn from a local hospital, while the external validation cohort was based on a U.S. database. Previous studies using the MIMIC-III dataset have demonstrated that the proportion of patients meeting various sepsis criteria may differ significantly across social determinants of health. When a universally trained model is applied across racial or ethnic subgroups, its performance in predicting mortality among septic patients may vary considerably^[5]^. Thus, the generalizability of the model across diverse populations deserves careful consideration.

Third, although the model was trained on 244 patients from a single institution, the authors did not detail key data characteristics such as heterogeneity, class imbalance, or the handling of missing data factors that critically affect model robustness. More importantly, the lack of external validation on independent multicenter datasets limits the translational relevance of the model. Without testing in varied clinical settings, it is difficult to assess its practical reliability, especially given differences in ICU care practices across hospitals.

Fourth, the predictive features appear to rely heavily on vital signs and laboratory measurements (e.g., total protein, hematocrit, red blood cell count, Injury Severity Score, and SOFA score). However, the temporal relationship between these features and the actual onset of sepsis is not clearly defined. Given that sepsis is a dynamic and time-sensitive condition, predictive modeling should specify the prediction window, lead time, and ideally incorporate time-aware approaches such as time-series modeling or survival analysis – none of which were addressed in the current study.

Fifth, while the authors trained complex machine learning models (LightGBM, eXGBM, and neural networks) using 244 patients (70% for training), the relatively small sample size raises concerns about model overfitting, particularly for neural networks. Without a clear description of the number of predictors or regularization strategies, the risk of overfitting remains high. According to TRIPOD and other modeling guidelines, such algorithms typically require larger datasets and stringent feature selection and regularization procedures to ensure generalizability^[6]^.

We appreciate the authors’ efforts in developing a potentially valuable tool for early sepsis prediction in trauma ICU patients. Nonetheless, the current model would benefit from enhanced methodological transparency and broader validation. We encourage the authors to further refine their modeling framework in future studies and to benchmark their tool against existing approaches.

## Data Availability

Not applicable.
